# Jejunal diverticulitis with misleading biochemical improvement: a diagnostic challenge

**DOI:** 10.1093/jscr/rjag722

**Published:** 2026-08-20

**Authors:** Shing Fung Oscar Miao, Priscilla Martin, Michael Donovan

**Affiliations:** Department of General Surgery, Sunshine Coast University Hospital, 6 Doherty Street, Birtinya, Sunshine Coast, Queensland 4575, Australia; Department of General Surgery, Sunshine Coast University Hospital, 6 Doherty Street, Birtinya, Sunshine Coast, Queensland 4575, Australia; School of Medicine and Dentistry, Griffith University, Sunshine Coast University Hospital, Sunshine Coast Health Institute, 6 Doherty Street, Birtinya, Queensland 4575, Australia; Department of General Surgery, Sunshine Coast University Hospital, 6 Doherty Street, Birtinya, Sunshine Coast, Queensland 4575, Australia

**Keywords:** jejunal diverticulitis, jejunoileal diverticular disease, small bowel diverticula, diagnostic laparoscopy, acute abdomen

## Abstract

Jejunal diverticulitis is a rare and often under-recognized cause of acute abdominal pain, frequently presenting with non-specific clinical, biochemical, and radiological findings. A 52-year-old female presented with acute abdominal pain and elevated inflammatory markers, but non-diagnostic imaging. Despite biochemical improvement with conservative management, her symptoms persisted. Diagnostic laparoscopy revealed jejunal diverticulitis with an incidental Meckel’s diverticulum requiring dual segmental small bowel resection. The postoperative course was uneventful. This case highlights the limitations of relying on biochemical and radiological findings in isolation. Persistent symptoms despite reassuring investigations should prompt consideration of operative exploration.

## Introduction

Jejunal diverticulitis is a rare condition, with an estimated prevalence of 0.1% [1]. Unlike colonic diverticular disease, it often presents with non-specific symptoms, and laboratory or radiological findings may be inconclusive, delaying diagnosis [2–4]. Computed tomography (CT) is the imaging modality of choice, though findings may be subtle or overlooked [4–6]. Consequently, diagnosis is often established intraoperatively [2]. We present a case of jejunal diverticulitis in a 52-year-old female with persistent symptoms despite equivocal investigations, highlighting the importance of clinical judgement in guiding timely operative intervention.

## Case report

A 52-year-old female presented to the emergency department with 2 days of worsening epigastric pain. The pain radiated to the back and was associated with nausea, vomiting, and subjective fevers. Her past medical and surgical history was unremarkable.

Examination demonstrated suprapubic tenderness without peritonism. Her temperature was 37.3°C, and she was borderline tachycardic with a heart rate of 102 bpm.

**Figure 1:**
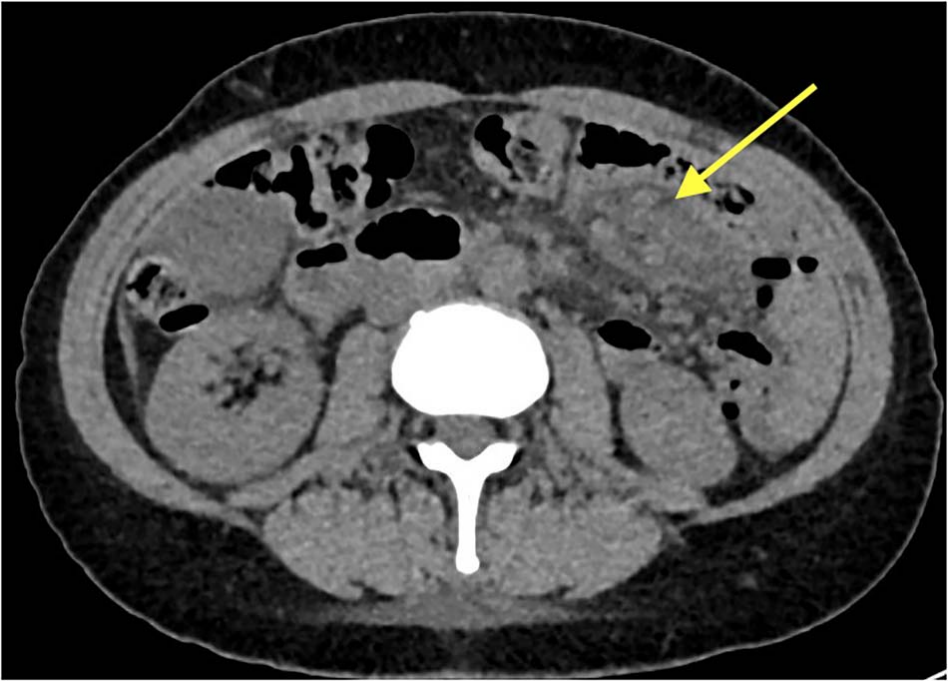
Initial portal venous phase contrast-enhanced CT of the abdomen on Day 1 demonstrating an indeterminate left mid-abdominal lesion (arrow), measuring ~39 mm in diameter, composed of mixed air and soft tissue attenuation. Associated small mesenteric lymph nodes are present. No free intraperitoneal air, free fluid, bowel obstruction, or radiological features of pancreatitis was identified. The differential diagnosis included an infected small bowel diverticulum and interloop abscess.

Initial investigations showed haemoglobin 142 g/L, white cell count (WCC) 9.8 × 10^9^/L with mild neutrophilia (8.37 × 10^9^/L), C-reactive protein (CRP) 202 mg/L, with normal lipase, renal/liver function, and lactate. CT of the abdomen and pelvis (portal venous phase) demonstrated an indeterminate abnormality within the left mid-abdomen, measuring ~39 mm, consisting of mixed air and soft tissue attenuation with adjacent small enhancing mesenteric lymph nodes (Fig. 1). No definitive cause for these findings was established. She was commenced on intravenous fluids, antibiotics, and restricted oral intake.

**Figure 2:**
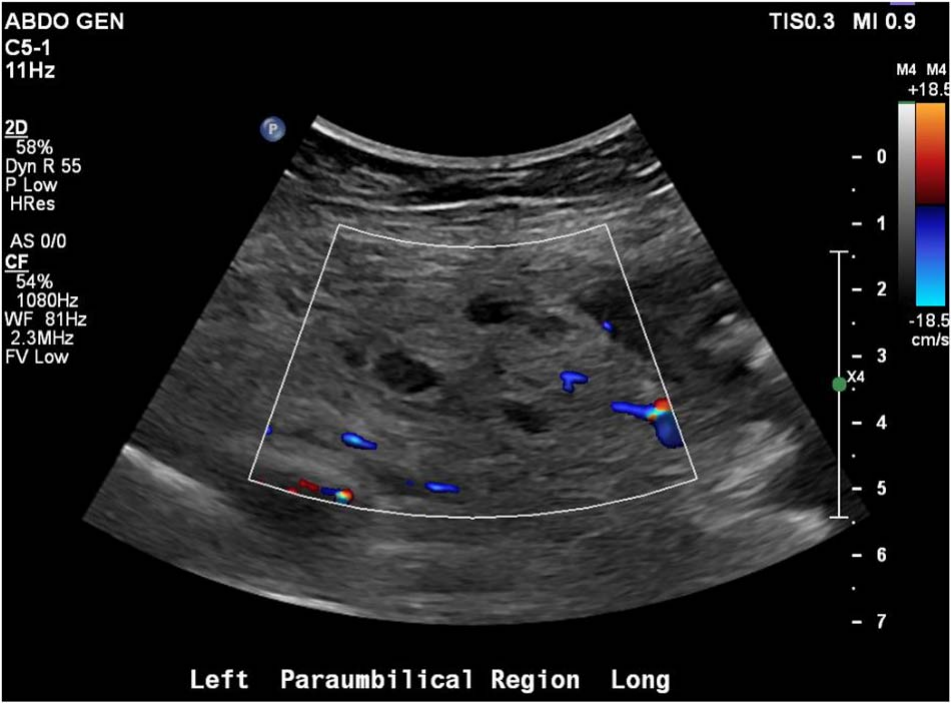
Longitudinal ultrasound image of the left paraumbilical region on Day 3 demonstrating a 40 × 28 × 36 mm non-peristaltic fluid- and gas-containing structure surrounded by echogenic inflamed mesentery and adjacent reactive mesenteric lymphadenopathy. Correlation with CT findings raised suspicion for jejunal diverticulitis with possible localized abscess formation.

**Figure 3:**
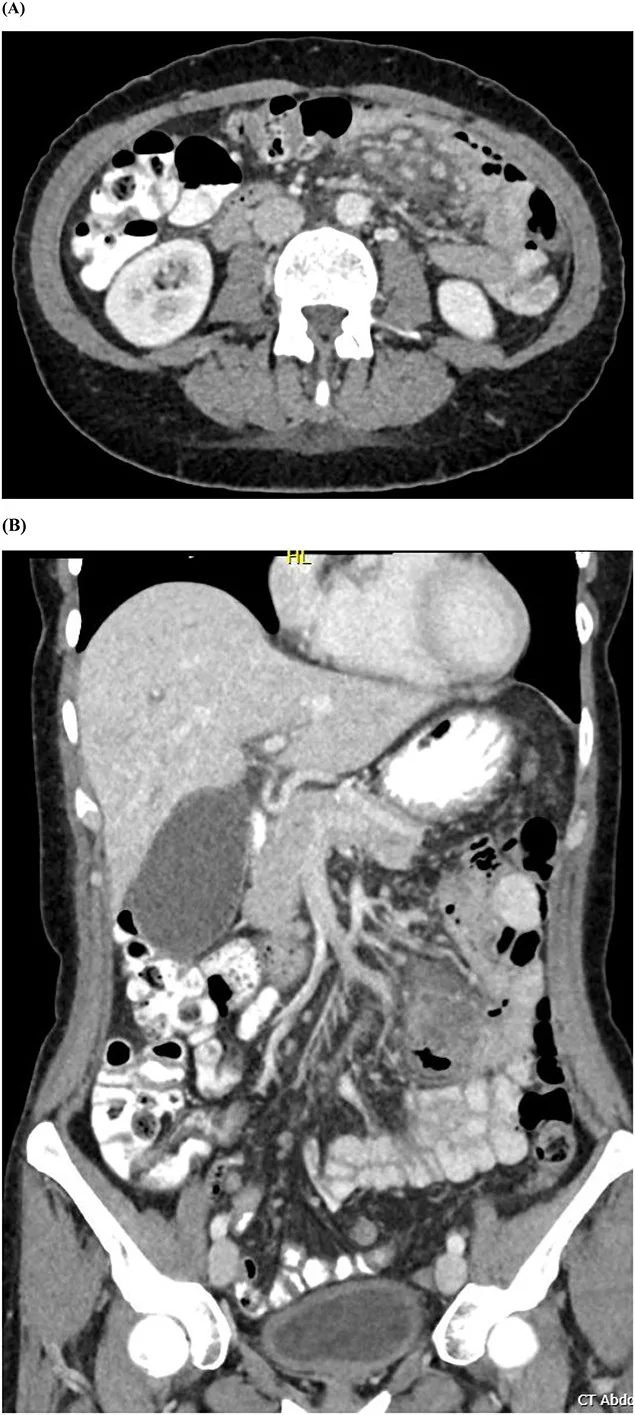
Oral and intravenous contrast-enhanced CT of the abdomen on Day 4. (A) Axial and (B) coronal images demonstrate a focally abnormal segment of jejunum in the left mid-abdomen with marked mural thickening, submucosal oedema, and surrounding mesenteric inflammatory change. No extraluminal contrast, free gas, or drainable collection was present. The findings were considered atypical and the preoperative differential diagnosis included jejunal diverticulitis and devascularized small bowel.

An abdominal ultrasound performed 48 hours later demonstrated an ill-defined gas and fluid collection in the left paraumbilical region, raising the possibility of jejunal diverticulitis (Fig. 2). Subsequent CT with oral and IV contrast the next day revealed abnormal jejunal loops in the left abdomen with marked submucosal oedema (Fig. 3). However, the findings remained non-specific, with differential diagnoses including atypical Meckel’s diverticulum, jejunal diverticulitis, or another cause of walled-off jejunal perforation.

Despite improving inflammatory markers (WCC 4.1 × 10^9^/L; CRP 39 mg/L), she had persistent pain, vomiting, and suprapubic tenderness, prompting diagnostic laparoscopy on Day 5. Laparoscopy identified a 15 cm inflamed proximal jejunal diverticular segment with interloop adhesions and fibrinous exudate, plus an incidental non-inflamed Meckel’s diverticulum distally. Two segmental small bowel resections with primary stapled anastomoses were performed.

The patient had an uncomplicated postoperative course and was discharged home on postoperative Day 6.

## Discussion

Acquired jejunoileal non-Meckel diverticula are rare, with an estimated prevalence of 0.03%–1% in the general population, markedly lower than that of colonic diverticulosis [7–9]. In contrast to colonic diverticula, these lesions are typically acquired pseudodiverticula arising along the mesenteric border, representing herniation of mucosa and submucosa through the muscularis layer [2]. They occur more frequently in males, with incidence increasing with age and peaking in the sixth and seventh decades of life [8, 10, 11]. Most patients remain asymptomatic or present with vague, non-specific gastrointestinal symptoms [12].

CT remains the imaging modality of choice; however, its diagnostic performance is variable, with reported accuracy ranging from 35% to 65% [10, 11]. This reflects difficulty distinguishing jejunal diverticulitis from other intra-abdominal pathology. Notably, diagnostic yield appears to correlate with disease severity, with reported accuracy of up to 80% in cases of perforation but as low as 36.7% in uncomplicated diverticulitis [9, 10]. The use of oral and rectal contrast may aid in delineating the origin of diverticula or may help rule out any suspected perforation [2].

Radiological assessment may therefore be inconclusive, and preoperative diagnosis is frequently missed [2]. Delayed or missed diagnosis carries significant risk, with complications such as perforation associated with mortality rates of up to 40% [13], emphasizing the importance of maintaining clinical vigilance.

A key diagnostic challenge lies in the inconsistency of clinical and biochemical findings. Normalization of inflammatory markers in our patient created a misleading impression of improvement despite ongoing symptoms. This discordance highlights an important limitation of relying solely on biochemical trends and underscores the need to prioritize the overall clinical picture.

Diagnostic laparoscopy plays a critical role in non-resolving scenarios, enabling both definitive diagnosis and therapeutic management [14]. In our case, early operative exploration was undertaken to mitigate the risk of progression to complications associated with jejunal diverticulitis, including perforation (2.1%–7%), mesenteric abscess formation, generalized peritonitis, intestinal obstruction (2.3%–4.6%), and diverticular haemorrhage (2%–8.1%) [15].

An additional intraoperative finding was an incidental Meckel’s diverticulum that appeared macroscopically non-inflamed. As the most common congenital anomaly of the gastrointestinal tract, Meckel’s diverticulum is present in ~2% of the population and is often identified incidentally during abdominal surgery [16, 17]. The management of incidentally discovered asymptomatic Meckel’s diverticulum remains controversial and should be individualized based on patient factors and intraoperative context [17]. In this case, concurrent resection was performed to mitigate the risk of future complications. Histopathological examination demonstrated a dilated Meckel’s diverticulum with attenuated muscularis propria and retained thick enteric contents; gross examination also suggested possible luminal narrowing, retrospectively supporting prophylactic resection.

Although the incidental Meckel’s diverticulum was macroscopically abnormal, histology showed no active inflammation or ectopic tissue, making it an unlikely contributor to the acute presentation. In contrast, histopathology of the resected jejunal segment confirmed ruptured diverticulitis with abscess formation, perforation, and acute peritonitis, supporting this as the primary pathological driver of the patient’s presentation.

This case highlights several important considerations. First, jejunal diverticulitis should remain an important differential in patients presenting with acute abdominal pain, even in the setting of non-specific laboratory and imaging findings. Second, biochemical improvement does not necessarily reflect clinical resolution, and persistent or evolving symptoms should prompt further investigation or early operative intervention. Finally, diagnostic laparoscopy remains an important tool in the evaluation of undifferentiated abdominal pain, facilitating timely diagnosis and definitive management when non-invasive investigations are inconclusive.

## Conclusion

Jejunal diverticulitis remains a rare and challenging diagnosis, particularly in the presence of non-specific imaging and discordant biochemical findings. This case highlights the limitations of relying solely on laboratory and imaging findings and underscores the importance of clinical judgement in guiding timely operative intervention in patients with persistent symptoms.

## Declaration

All the authors declare that our information provided here is accurate to the best of our knowledge.
